# Lessons in hand washing: what we should know?

**DOI:** 10.1186/2047-2994-4-S1-P283

**Published:** 2015-06-16

**Authors:** LO Egwari, OO Ayepola, MI Oniha, GI Olasehinde

**Affiliations:** 1 Biological Sciences, Covenant University, Ota, Nigeria

## Introduction

Most communicable diseases are transmitted by hand; from nosocomial to community acquired diseases. Consequently, hand washing is strictly advocated in hospital, eateries and public places where contact by hand is frequent.

## Objectives

The effect of hand washing in reducing the microbial load of the hand in line with specified hand washing guidelines was determined.

## Methods

Two categories of volunteers, supervised and un-supervised hand washing were studied and percentage of subjects in each category showing microbial reduction by 50%, 80% and 99% was recorded. Swab samples were collected with moistened swabs before and after hand washing. Samples were cultured and assessed qualitative and quantitative.

## Results

Supervised washed hands yielded 93, 75 and 38 percent reduction of microbial load by 50%, 80% and 99% respectively contrast reduction by similar proportion by 68, 15, and 7 percent in the un-supervised washed hands. The difference in reduction of bacterial load in supervised and unsupervised groups was significant (p>0.01). An increase in bacterial load was seen after hand washing in 18-26% of volunteers with higher extreme occurring in unsupervised hand wash. Bacterial that persisted or increased in number following hand washing were *Pseudomonas aeruginosa, Klebsiella*, spp, *Enterobacter* spp *and Escherichia coli*. There was no gender preference but, persons with long nails yielded more microbiota in type and population.

## Conclusion

Adequate hand washing and the use of germicidal soap may represent the rule in preventing transmission of diseases communicable by hand.

## Disclosure of interest

None declared.

